# Pemafibrate, a selective PPARα modulator, prevents non-alcoholic steatohepatitis development without reducing the hepatic triglyceride content

**DOI:** 10.1038/s41598-020-64902-8

**Published:** 2020-05-08

**Authors:** Yusuke Sasaki, Masato Asahiyama, Toshiya Tanaka, Shogo Yamamoto, Kentaro Murakami, Wakana Kamiya, Yoshihiro Matsumura, Tsuyoshi Osawa, Motonobu Anai, Jean-Charles Fruchart, Hiroyuki Aburatani, Juro Sakai, Tatsuhiko Kodama

**Affiliations:** 1 Department of Nuclear Receptor Medicine, Laboratories for Systems Biology and Medicine (LSBM) at the Research Center for Advanced Science and Technology (RCAST), The University of Tokyo, Tokyo, 153-8904 Japan; 2 Pharmaceutical Division, Kowa Company, Ltd., Tokyo, 189-0022 Japan; 3 Genome Science Division, Laboratories for Systems Biology and Medicine (LSBM) at the Research Center for Advanced Science and Technology (RCAST), The University of Tokyo, Tokyo, 153-8904 Japan; 4 Division of Metabolic Medicine, Laboratories for Systems Biology and Medicine (LSBM) at the Research Center for Advanced Science and Technology (RCAST), The University of Tokyo, Tokyo, 153-8904 Japan; 5 Division of Integrative Nutriomics and Oncology, Laboratories for Systems Biology and Medicine (LSBM) at the Research Center for Advanced Science and Technology (RCAST), The University of Tokyo, Tokyo, 153-8904 Japan; 6 R3i Foundation, Picassoplatz 8, 4010, Basel, Switzerland; 7 Division of Molecular Physiology and Metabolism, Tohoku University Graduate School of Medicine, Sendai, Miyagi 980-8575 Japan

**Keywords:** Drug discovery, Diseases, Endocrinology, Molecular medicine

## Abstract

Non-alcoholic steatohepatitis (NASH) is characterized by macrovesicular steatosis with ballooning degeneration of hepatocytes, diffused lobular inflammation, and fibrosis. PPAR ligands are promising therapeutic agents in NASH; accordingly, we evaluated the effects of the first clinically available selective PPARα modulator, pemafibrate. We found that pemafibrate improves F4/80-positive macrophage accumulation, ballooning degeneration of hepatocytes, and the non-alcoholic fatty liver disease (NAFLD) activity score without affecting triglyceride (TG) accumulation in the liver of a mouse model of NASH (STAM). A global gene expression analysis indicated that pemafibrate enhances TG hydrolysis and fatty acid β-oxidation as well as re-esterification from dihydroxyacetone 3-phosphate and monoacylglycerol to TG. These changes are accompanied by the induction of genes involved in lipolysis and lipid droplet formation, along with an increased number and reduced size of lipid droplets in pemafibrate-treated livers. Pemafibrate reduced the expression of the cell adhesion molecule *Vcam-1*, myeloid cell markers, and inflammation- and fibrosis-related genes in STAM mice. Furthermore, pemafibrate significantly reduced *VCAM-1* expression induced by high glucose in cultured human umbilical vein endothelial cells. These results suggest that pemafibrate prevents NASH development by reducing myeloid cell recruitment via interactions with liver sinusoidal endothelial cells, without altering hepatic TG accumulation.

## Introduction

Non-alcoholic fatty liver disease (NAFLD) is the most common cause of chronic liver disease and is closely linked to metabolic syndrome. Current estimates indicate that up to 30% of the general population is affected by NAFLD in industrialized countries^1^. Furthermore, 5–10% of patients with NAFLD can progress to non-alcoholic steatohepatitis (NASH), a more severe form of NAFLD that is broadly defined by the presence of steatosis and inflammation with ballooning, regardless of fibrosis, and eventually to cirrhosis and hepatocellular carcinoma^2–4^.

Hepatic TG accumulation has been suggested to play a central role in NASH development, but additional factors, such as insulin resistance, oxidative stress, ER stress, and mitochondrial dysfunction may also be involved^5,6^. However, the mechanisms underlying the pathogenesis of NASH for the subset of patients with steatosis has not been clarified. Additionally, no therapeutic agent has been approved for NASH. Therefore, there is an urgent need to develop an effective therapeutic approach for NASH.

Liver fatty acids and TG metabolism are tightly regulated by a balance of *de novo* lipogenesis (DNL), glyceroneogenesis, VLDL assembly and secretion, lipolysis, and fatty acid oxidation (FAO) at the transcriptional and post-transcriptional levels^7,8^. DNL is mainly transcriptionally regulated by sterol regulatory element binding protein 1c (SREBP1c) and carbohydrate response element binding protein (ChREBP), which are activated by increases in insulin signaling and glucose levels, respectively. PPARα induces hepatic FAO genes in the fasting state. Several studies have indicated that impaired PPARα function and FAO are major determinants of NASH development^9,10^. Therefore, PPARα ligands are considered candidate therapeutic agents for NASH.

Pemafibrate (also known as K-877), approved in Japan, is expected to replace fibrates as the first clinically available selective PPARα modulator (SPPARMα) to improve dyslipidemia and reduce macro- and micro-vascular complications^11,12^. Pemafibrate has greater PPARα activation potency than those of other fibrates with a lower EC_50_ value and a high degree of subtype selectivity (>2,000-fold subtype selectivity)^13^. In preclinical and clinical studies, pemafibrate shows greater plasma TG lowering and HDL-cholesterol elevating effects than those of other fibrates on the market^14,15^. We have reported that pemafibrate induces a series of PPARα target genes involved in TG hydrolysis, fatty acid uptake, fatty acid β-oxidation, and ketogenesis in the liver, supporting its ability to reduce plasma TG^13^. Recently, Honda *et al*. reported that pemafibrate treatment improves obesity, dyslipidemia, liver dysfunction, and the pathological condition of AMLN diet-induced NASH model^16^. While AMLN model showed histologic and metabolic features of human NASH with diffuse fibrosis, this model did not induce cirrhosis, hyperglycemia and hypertriglyceridemia^17^. In this study, we tested the therapeutic potential of pemafibrate in STAM NASH model mice, which showed diabetes-based NASH-HCC within 20 weeks of age^18^.

## Results

### Pemafibrate prevents NASH development

To investigate the protective effects of pemafibrate against NASH, we used the STAM mouse model (induced NASH in male C57BL/6J strain)^18^. STAM mice showed significant hyperglycemia, hypertriglyceridemia, higher NEFA and ALT levels, lower body weights, and higher liver weights and relative liver weights than those of normal C57BL/6J mice (Table 1). Obvious difference in food intake was not observed between STAM control (2.70 g/day) and pemafibrate-treated group (2.65 g/day). Pemafibrate effectively reduced serum TG and NEFA levels but did not alter the serum glucose, AST, and ALT levels. In addition, pemafibrate significantly increased liver weight and relative liver weight, which is a well-known effect in response to PPARα stimulation in rodents^19^.

**Table 1:** Effects of pemafibrate on body and liver weight, biochemical parameters in the serum, immunohistochemical analysis, and NAS.

	Normal			STAM					
				Vehicle			Pemafibrate		
Body weight (g)	23.6	±	0.3**	17.4	±	0.8	18.4	±	0.5
Liver weight (g)	1.0	±	0.0**	1.3	±	0.0	1.8	±	0.0**
Relative liver weight (g liver/100 g Body weight)	4.3	±	0.1**	7.6	±	0.4	9.7	±	0.3**
AST (U/l)	104.2	±	6.4	229.2	±	79.5	173.3	±	7.5
ALT (U/l)	27.8	±	2.0*	90.7	±	35.1	75.5	±	6.2
Glucose (mg/dL)	140.4	±	8.6**	491.1	±	22.7	511.1	±	21.1
NEFA (mEq/l)	0.7	±	0.0**	2.3	±	0.4	1.1	±	0.1**
Triglyceride (mg/dL)	105.4	±	7.5**	867.0	±	101.6	270.2	±	49.9**
Total Cholesterol (mg/dL)	80.5	±	4.5**	178.7	±	9.0	191.0	±	9.8
F4/80 positive area (%)	2.4	±	0.1**	5.9	±	0.6	4.3	±	0.3*
ER-TR7 positive area (%)	1.6	±	0.1*	3.1	±	0.6	2.2	±	0.3
Sirius red positive area (%)	0.3	±	0.0**	0.5	±	0.1	0.4	±	0.0
Steatosis	0.0	±	0.0**	1.8	±	0.3	1.8	±	0.2
Inflammation	0.0	±	0.0**	1.8	±	0.2	1.7	±	0.2
Ballooning	0.0	±	0.0**	1.5	±	0.2	0.3	±	0.2**
NAS	0.0	±	0.0**	5.2	±	0.6	3.8	±	0.5*

AST: aspartate aminotransferase, ALT: alanine aminotransferase, NEFA: non-esterified fatty acid, NAS: NAFLD activity score, n = 6 animals per group. Error bars show s.e.m. *P < 0.05; **P < 0.01: Significantly difference from STAM control group by Dunnett’s multiple comparison test.

**Figure 1:**
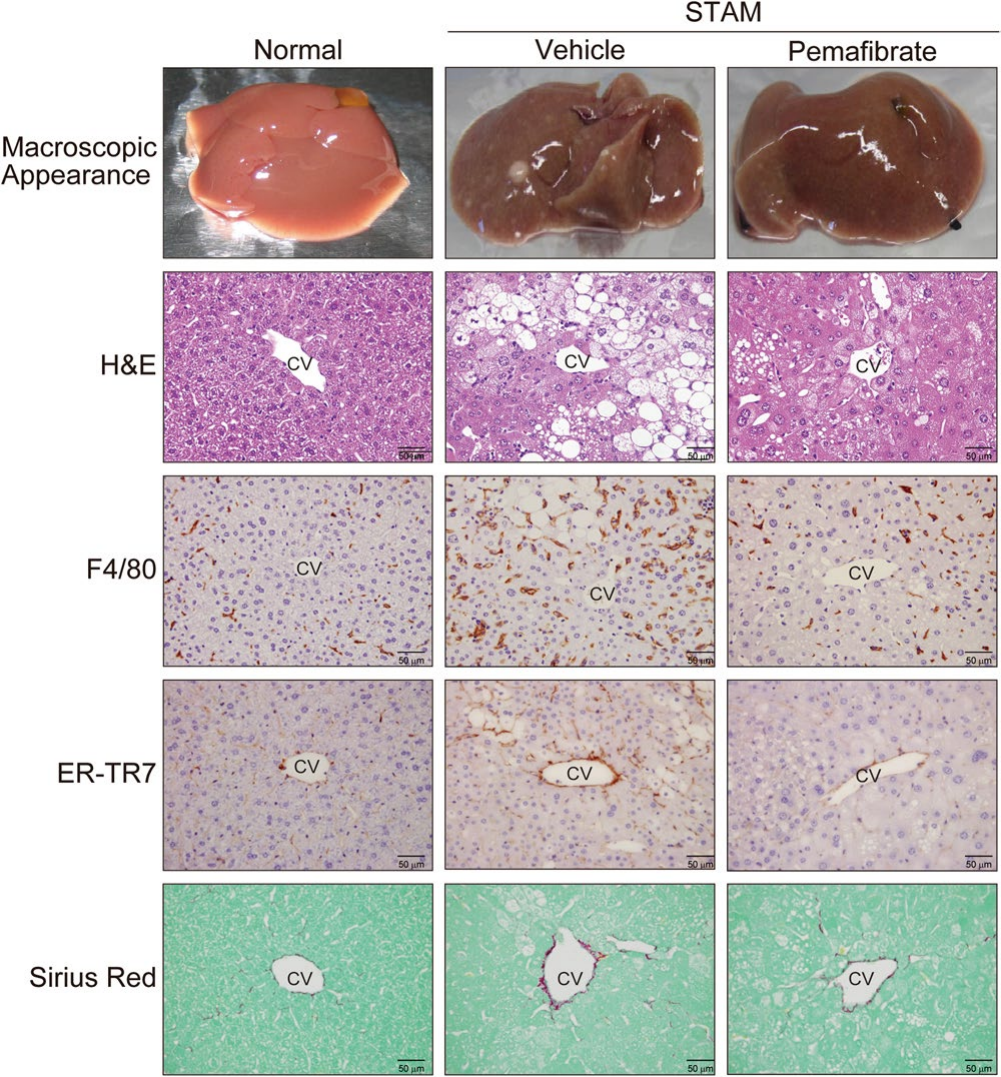
Pemafibrate improves macrovesicular steatosis and F4/80 positive cell accumulation in STAM mice liver. Representative gross morphology of liver, H&E stained, F4/80 stained, ER-TR7 stained, and Sirius-red stained liver section from normal, vehicle, and pemafibrate treated STAM mice.

Representative macroscopic images and microscopic H&E-stained liver sections from STAM control mice exhibited liver nodules, macro- and micro-vesicular lipid accumulation, inflammatory cell infiltration, and ballooning degeneration of hepatocytes, unlike in normal mice (Fig. 1). Pemafibrate-treated mice showed less macrovesicular lipid accumulation, less ballooning degeneration, and a significantly lower NAFLD activity score (NAS)^20^ than those of STAM control mice (Table 1).

ER-TR7 and Sirius Red staining demonstrated pericellular collagen deposition and fibrosis around central veins in STAM control mice (Fig. 1 and Table 1). Pemafibrate did not significantly improve the ER-TR7- and Sirius Red-positive areas. Marked lobular F4/80^+^ macrophage accumulation was observed in the STAM control livers. The pemafibrate-treated group showed a significantly smaller F4/80^+^ area than that of the STAM control group (Fig. 1 and Table 1).

### Pemafibrate induces TG synthesis from DHAP and glycerol and re-esterification of TG in STAM mouse livers

**Figure 2:**
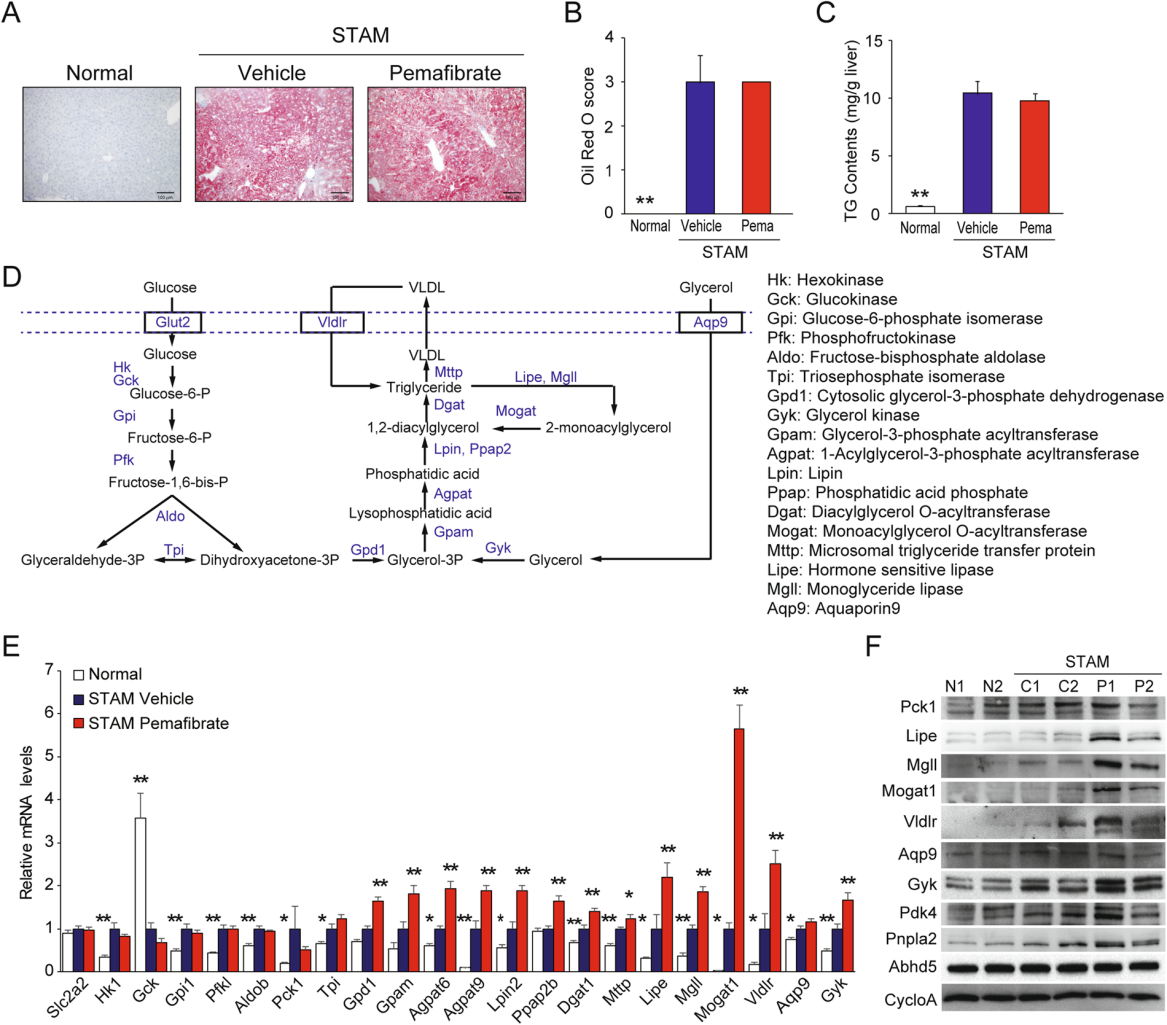
Pemafibrate induces TG synthesis in STAM mice liver. (**A**) Oil-Red-O stained liver section from normal, vehicle, and pemafibrate treated STAM mice. (**B**) Quantification of oil-red-O staining (n = 6). (**C**) TG contents in liver (n = 6). (**D**) Schematic representation of the glycolytic and TG synthesis pathways in the liver (**E**) qPCR validation of glycolytic and TG synthesis pathways (n = 6). (**F**) Immunoblots for TG metabolism-related proteins in liver extracts. Error bars show s.e.m. *P < 0.05; **P < 0.01: Significantly difference from STAM control group by Dunnett’s multiple comparison test.

Although pemafibrate effectively reduced macrovesicular steatosis, it did not improve the steatosis score (Table 1). To better understand the effect of pemafibrate, we used Oil Red-O staining to evaluate the TG concentration in the liver (Fig. 2A–C). The STAM control group showed a significantly increased area of fat deposition and higher TG content in the liver. There were no significant differences in the area of fat deposition and TG content between the STAM control group and pemafibrate-treated group (Fig. 2A–C).

To verify the effect of pemafibrate on STAM mouse livers, we performed a comprehensive transcriptome analysis by RNA-seq using liver tissues collected from normal, STAM control, and pemafibrate-treated STAM mice. We identified 187 up-regulated and 477 down-regulated genes in the pemafibrate-treated compared with the STAM control group by our stringent criteria (Supplementary Table 1). Using the web-based tool FuncAssociate 2.1 (http://llama.mshri.on.ca/funcassociate/) for a GO analysis, the upregulated genes were enriched for functions related to lipid metabolic processes and the downregulated genes were enriched for immune system processes (Supplementary Table 2). In fact, PPARα–regulated FAO-related genes were significantly induced in the pemafibrate-treated group (Supplementary Fig. 1).

To determine why pemafibrate did not reduce TG accumulation in STAM mouse livers, we further investigated its effect on lipid and carbohydrate metabolic gene expression. The expression levels of genes related to TG hydrolysis, fatty acid uptake, fatty acid activation, fatty acid binding, peroxisomal and mitochondrial oxidation, and ketogenesis were higher in the STAM control group than in the normal group (Supplementary Figs. 1 and 2). Pemafibrate apparently induced the expression of these genes. In particular, pemafibrate treatment resulted in the greatest increase in *Pdk4* expression, suggesting that it mediates the suppression of glucose oxidation and preferential activation of fatty acid oxidation (Supplementary Figs. 1 and 2).

Increased glucose uptake in hepatocytes promotes glycolysis and lipogenesis to generate TG. In eukaryotes, the glycerolipid synthesis pathway (glyceroneogenesis) and the monoacylglycerol pathway play central roles in TG synthesis (Fig. 2D)^21,22^. The STAM control group showed higher levels of glycolysis-related gene expression than those in the normal group (Supplementary Fig. 3). In addition, we found that levels of *Pck1*, which encodes a key enzyme that catalyzes the conversion of oxaloacetate to phosphoenolpyruvate^23^, were increased in the STAM group, indicating that enhanced glyceroneogenesis contributes to lipogenesis in STAM mouse livers (Fig. 2E,F and Supplementary Fig. 3). Moreover, re-esterification of 2-monoacylglycerol was induced in STAM control livers in addition to simultaneous TG uptake and lipolysis. Pemafibrate did not influence glycolysis and *Pck1* expression but significantly induced a series of genes involved in TG synthesis from DHAP and glycerol (Fig. 2E,F). Pemafibrate had the greatest effect on *Mogat1*, which has key roles in TG re-esterification from monoacylglycerols and diacylglycerols generated by TG hydrolysis^24^ in STAM mouse livers (Fig. 2E,F). These results suggest that pemafibrate enhances TG synthesis from DHAP and glycerol and the re-esterification of glycerol generated by TG hydrolysis in STAM mouse livers.

### Pemafibrate increases microvesicular lipid droplets in STAM mouse livers

**Figure 3:**
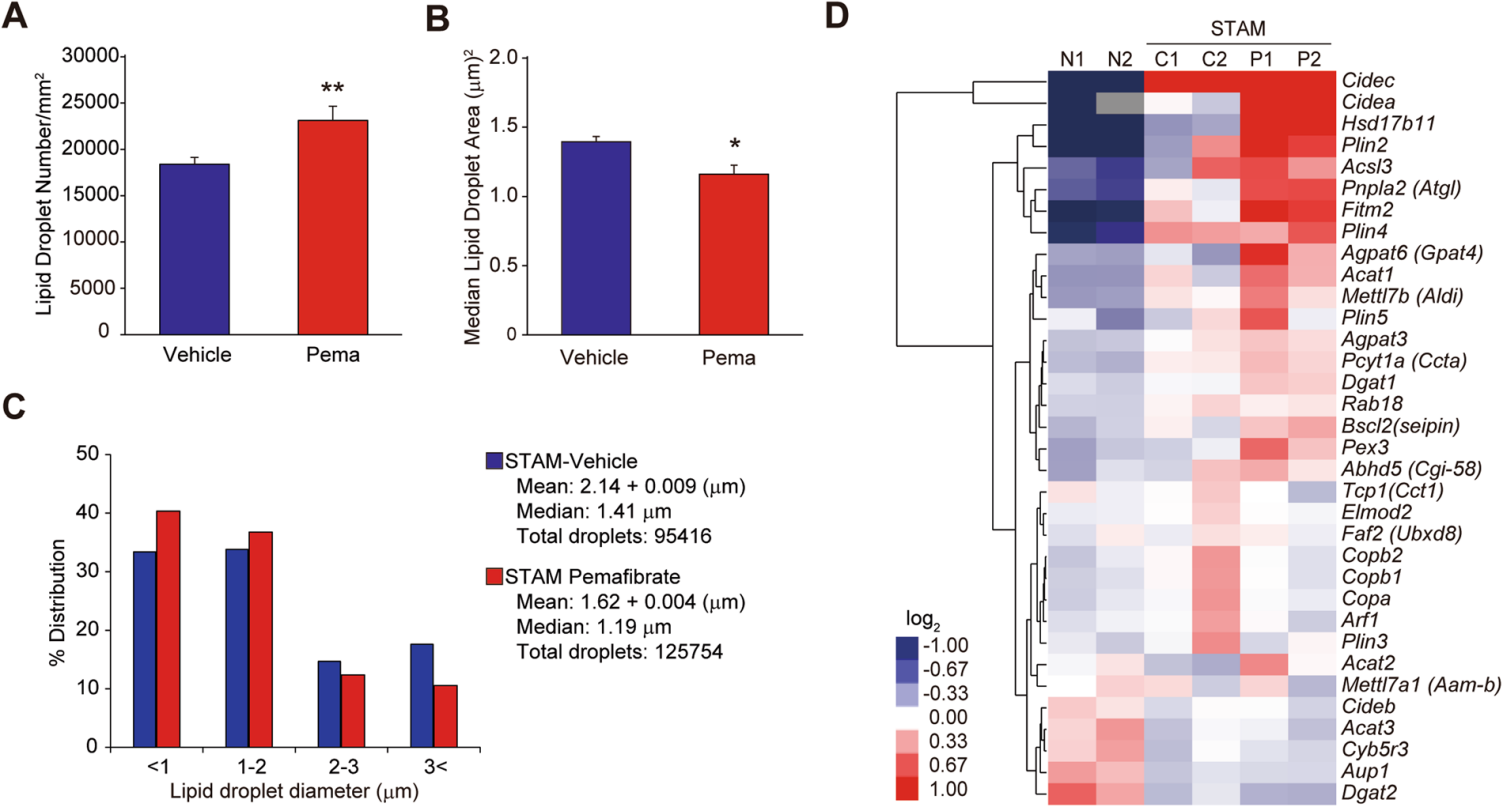
Pemafibrate induces lipid droplets formation. (**A**) Quantification of lipid droplet number of vehicle and pemafibrate treated STAM mice. (**B**) Median lipid droplet area of vehicle and pemafibrate treated STAM mice. (**C**) Investigation of hepatic lipid droplet sizes in vehicle and pemafibrate treated STAM mice. (**D**) Heatmap of hierarchical clustering of LDAP and formation-related genes. Error bars show s.e.m. *P < 0.05; **P < 0.01: Significantly difference from STAM control group by Bonferoni’s multiple comparison test.

Because pemafibrate improved macrovesicular steatosis without reducing hepatic TG accumulation (Fig. 2), we next evaluated lipid droplet counts and size distributions. Pemafibrate treatment increased the droplet number and decreased the lipid droplet area (Fig. 3A,B). Pemafibrate increased the percentage of cells expressing small lipid droplets (<1 μm) from 34% in the STAM control to 40% and decreased large lipid droplets (>3 μm) from 18% in the STAM control to 11% (Fig. 3C). Lipid droplets consist of an inner core of neutral lipids including TG and sterol esters, a phospholipid monolayer, and lipid droplet-associated proteins (LDAPs)^25,26^. LDAPs influence lipid droplet function and dynamics; accordingly, we evaluated the effect of pemafibrate on LDAPs expression (Fig. 3D). The STAM control group showed increases in the expression of genes related to lipid droplet inner core lipid synthesis (*Agpat6*, *Dgat1*, and *Acat1*), formation (*Agpat6*, *Acsl3*, and *Plin2*), lipolysis (*Pnpla2*, *Hsd17b11*, and *Abhd5*), budding (*Fitm2* and *Bscl2*), expansion (*Arf1*, *Copa*, *Copb1*, *Copb2*, *Rab18*, and *Elmod2*), and fusion (*Cidea* and *Cidec*). Pemafibrate further induced the *Agpat6*, *Plin2*, *Pnpla2*, *Hsd17b11*, *Fitm2*, *Cidea*, and *Cidec* expression. Importantly, changes in *Pnpla2* mRNA expression level were reflected at the protein level in mice liver (Fig. 2F).

### Pemafibrate reduces macrophage interactions with liver sinusoidal endothelial cells

**Figure 4:**
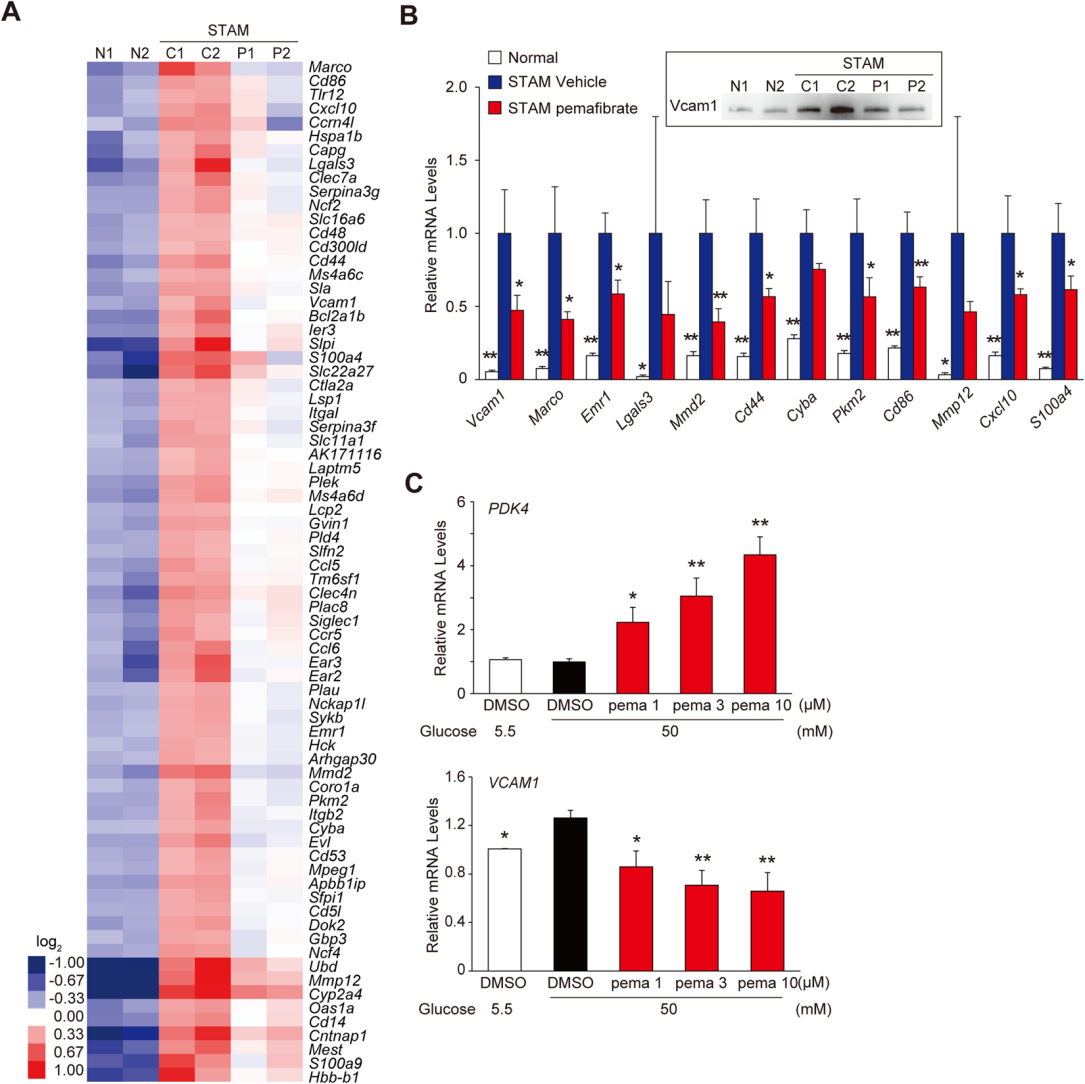
Pemafibrate improves inflammatory genes expression in STAM mice liver. (**A**) Heatmap showing changes in expression of selected 74 genes. (**B**) qPCR validation of myeloid cell marker and inflammatory genes in the liver (n = 6). Immunoblot of Vcam1 protein is shown in upper boxed panel. Error bars show s.e.m. *P < 0.05; **P < 0.01. (**C**) HUVECs were cultured and treated with DMSO or pemafibare for 24 h in the presence of 50 mM Glucose. qPCR validation of *PDK4* and *VCAM1* expression (n = 3). Error bars show s.e.m. *P < 0.05; **P < 0.01: Significantly difference from STAM control group by Dunnett’s multiple comparison test.

We further evaluated 74 of 473 genes that fulfilled more stringent criteria (FPKM of STAM control ≥3; STAM control/normal ratio ≥3; pemafibrate/STAM control ratio <2^−0.6^), as presented in a heat map in Fig. 4A. Livers from the STAM control group showed enhanced macrophage recruitment and inflammation. They expressed a number of polarization markers, including *Marco*, *Emr1*, *Mmd2*, *Cd44*, *Pkm2*, and *Cd86*. Oxidative stress and inflammatory responses have critical roles in NASH development. The NADPH oxidase components *Cyba* and *Ncf4* and inflammatory factors *Mmp12*, *Cxcl10*, *S100a4*, and *Lgals3* were highly induced in the STAM control mice and were significantly reduced in the pemafibrate-treated group. Resident tissue macrophages and monocyte-derived macrophages are important in chronic inflammatory processes. During inflammation, the induction of vascular cell adhesion molecule- 1 (VCAM-1) and CD31 is reported to promote the transendothelial migration of leucocytes^27^. Indeed, our transcriptome analysis indicated that *Vcam1* levels are elevated in STAM control livers and are significantly reduced by pemafibrate treatment (Fig. 4B). These data suggested that pemafibrate prevents inflammatory monocyte recruitment and differentiation.

Dysfunction of liver sinusoidal endothelial cells (LSECs) and the recruitment of inflammatory cells promote liver injury and inflammation^27,28^. In particular, high levels of glucose induce adhesion molecules, the transendothelial migration of monocytes, and monocyte-endothelial adhesion in human coronary artery endothelial cells and HUVECs^29,30^. We asked whether pemafibrate treatment prevents high-glucose-induced VCAM-1 expression in HUVECs. In accordance with previous reports, high glucose treatment induced *VCAM1* expression in HUVECs (Fig. 4C). Pemafibrate dose-dependently reduced *VCAM1* expression. This effect paralleled the induction of the well-known PPAR target *PDK4*. These data suggest that PPARα activation by pemafibrate in LSECs is pivotal for the prevention of NASH in STAM mouse livers.

## Discussion

During NAFLD/NASH progression, lipotoxicity and interactions between hepatic myeloid cells and LSECs play pivotal roles^31^. Therefore, the STAM control of lipid flux to prevent hepatic TG accumulation and reduce hepatic inflammation is a potential therapeutic strategy for the prevention and reversion of NASH. We hypothesized that the activation of the SPPARMα pemafibrate would reduce hepatic TG accumulation and have therapeutic effects in NASH. Using the STAM mouse model, we found that therapeutic intervention with pemafibrate significantly improved the histological severity (ballooning and NAS) as well as inflammatory and fibrosis marker gene expression, without altering the hepatic TG content. A comprehensive transcriptome analysis revealed that pemafibrate decreases numerous immune-responsive genes in STAM mouse livers. In addition, pemafibrate reduced immune cell infiltration in diseased liver and high glucose-induced *VCAM1* expression in HUVECs. These results suggest that PPARα activation by pemafibrate has therapeutic potential for NASH. In addition, the results emphasize the necessity of combinations of two or more drugs to modulate hepatic lipid flux and inflammation.

Under high fat and/or carbohydrate intake, lipogenesis is stimulated and excess fat is stored as TG. Excess TG accumulation in the liver results in hepatic steatosis, causing cellular lipotoxicity, fibrosis, and NASH^8^. This arises from an imbalance between TG accumulation (i.e., FA uptake and DNL) and removal (i.e., lipolysis and VLDL secretion)^32^. As indicated in Table 1, the STAM model was characterized by hyperglycemia and reduced body weight with a nearly complete loss of insulin production. Therefore, SREBP1c-mediated DNL is unlikely to contribute to TG accumulation in this model. In fact, a consistent increase in DNL was not observed in STAM model livers despite a substantial increase in genes encoding glycolytic enzymes (Supplementary Fig. 3). TG acquisition also involves fatty acid esterification pathways. The process of fatty acid esterification into TG initiates from the transfer of Acyl-CoA to the hydroxyl groups of glycerol-3-phosphate (G3P). G3P is derived from glucose via glycolysis, plasma glycerol, and *de novo* synthesis from pyruvate, lactate, or amino acids by glyceroneogenesis (a truncated version of gluconeogenesis)^8^. Recently, glyceroneogenesis has been recognized as an important pathway for G3P supply^33^ and a therapeutic target. Several reports have indicated that the liver utilizes mainly glycerol from glyceroneogenesis for TG synthesis in humans and rodents^23,34,35^. Our transcriptome analysis suggests that glyceroneogenesis is a G3P source for TG synthesis, as evidenced by the induction of higher levels of *Pck1* (a rate-limiting enzyme in glyceroneogenesis) than *Aqp9* or *Gyk*. In addition, we observed the substantial induction of genes related to TG hydrolysis (*Lipe* and *Mgll*) and re-esterification (*Mogat1*). Therefore, the enhancement of glyceroneogenesis and TG re-esterification are likely to play significant roles in TG accumulation in STAM mouse livers. PPARα is a well-established nutrient sensor for the induction of FAO and gluconeogenesis in the fasted liver^36^. In this study, PPARα activation by pemafibrate further induced FAO, TG hydrolysis, and re-esterification but did not influence glycolysis or the glyceroneogenesis rate-limiting enzyme *Pck1* (Fig. 2E,F). Interestingly, various genes involved in TG synthesis from DHAP were significantly induced in pemafibrate-treated STAM mouse livers (Fig. 2E). As shown in Fig. 2F and Supplementary Figs. 1 and 2, pemafibrate had the greatest effect on *Pdk4*. Since Pdk4 is an inhibitor of glucose oxidation, PPARα activation by pemafibrate preferentially enhances fatty acid utilization for FAO and ketogenesis in the liver^11,13^. In contrast, our results suggested that the blocking of glucose oxidation by pemafibrate has a compensatory effect by enhancing the conversion of the excess glycolytic intermediate DHAP to TG synthesis in STAM mouse livers. Therefore, these results indicated that PPARα activation by pemafibrate simultaneously induces TG hydrolysis, FAO, TG synthesis from DHAP, and TG re-esterification under hyperglycemia and hyperlipidemia and thereby could not reduce TG accumulation in the STAM mouse model.

Macrovesicular steatosis (large droplet steatosis) is associated with the development of lobular inflammation and fibrosis in NASH^8^. Recent several reports have shed light on lipid droplet biology and its role in the pathogenesis of steatosis. A core of neutral lipids (i.e. TG and sterol esters) is surrounded by a phospholipid monolayer and LDAPs, and these have a key role in lipid droplet biology^37^. Neutral lipids of lipid droplets (i.e., TG and cholesterol ester) are synthesized by DGATs and ACATs residing primarily in the ER^38^. In addition, perilipins, seipin (encoded by *Bscl2*), and FITM2 are involved in lipid droplet formation and budding to the cytoplasm^25,26^. Furthermore, large lipid droplets are formed by coalescence or through cell death-inducing DEF45-like effector (CIDE) family protein-mediated enhancement of neutral lipids diffusion from one lipid droplet to another^39^. In this study, pemafibrate resulted in microvesicular steatosis with an increased number of small lipid droplets compared with those in STAM mouse livers. Our transcriptome analysis indicated that pemafibrate enhances inner core lipid synthesis and formation (*Agpat6* and *Dgat1*), budding (*Fitm2*), and fusion (*Cidea*, and *Cidec*) genes expression (Fig. 3D). Recent evidence suggests that DGAT1 is responsible for the esterification of excess fatty acids^40^ and prevent lipotoxicity^41^. In addition, several reports indicated that CIDEA and CIDEC are both involved in the formation of large lipid droplet and induced in the hepatic steatosis^42^. As seen in adipocytes, the CIDE family proteins have been proposed to have a similar role in hepatocytes to promote lipid droplet formation^43^. CIDEA and CIDEC are strongly expressed in brown adipocytes and white adipocytes, and there multiolocular small lipid droplet formation by CIDEA and unilocular lipid droplet formation by CIDEC are linked with lipolysis and lipid storage, respectively. Thus, pemafibrate-induced *Cidea* expression may modulates lipid droplet size and basal lipolysis. In fact, pemafibrate also induced *Pnpla2* which catalyze TG hydrolysis on the lipid droplet surface to generate diacylglycerol and free fatty acids^44^. These results suggest that pemafibrate induces DGAT1-dependent TG re-esterification, initial lipid droplet formation and budding may explain the increased number of lipid droplets. Moreover, pemafibrate-induced *Cidea* and *Pnpla2* expression may explain a part of the prevention of larger lipid droplet formation in STAM mouse livers.

Lipotoxicity of hepatocytes initiate inflammatory cascades via the secretion of CXCL10-enriched extracellular vesicles, thereby inducing macrophage chemotaxis^45^. In addition, hepatic macrophage infiltration under steatohepatitis is induced by the interaction between myeloid cells and LSECs^46^. This interaction is mediated by cell adhesion molecules, such as VCAM1 and ICAM1, on LSECs^47–49^. In response to exposure to excess fatty acids, Kupffer cells and recruited macrophages undergo polarization to the M1 phenotype, characterized by increased production of cytokines, such as TNFα, IL6, IL-1β, and CCL5^47,49,50^. These cytokines further activate hepatic stellate cells (HSCs), which in turn secrete proinflammatory cytokines, such as IL34, CCL5, and CCL20^51–53^. Furthermore, activated HSCs cause LSEC capillarization and ROS production^46,54^. Thus, a feed-forward activation loop of macrophages, HSCs, and LSECs plays a critical role in the liver fibrosis. Importantly, recent findings suggest that LSEC injury precedes hepatic inflammation and fibrosis in NASH^31^. In this study, we found that pemafibrate reduces the expression of polarization, inflammation, and fibrosis marker genes along with *Vcam1* in STAM mouse livers (Fig. 4A,B). In addition, pemafibrate reduced high glucose-induced *VCAM1* expression in HUVECs (Fig. 4C). Therefore, although mechanisms underlying LSEC dysfunction are still unknown, anti-inflammatory effects of pemafibrate may be explained its direct and/or indirect protective effect on LSECs.

The STAM model of NASH^18^ is characterized by the nearly complete loss of pancreatic insulin production with severe hyperglycemia indicating this model represents diabetes-based NASH patients. It may not completely reflect the hepatic nutrient status of human NASH livers because human NASH is closely linked to type 2 diabetes. In fact, 3-fold higher levels of DNL have been detected in patients with NAFLD than in healthy individuals^55^, but apparent induction of DNL was not observed in STAM mouse liver. Therefore, additional studies using other NASH models with obesity and insulin resistance are warranted to elucidate the mechanism by which pemafibrate effects NAFLD/NASH. Interestingly, pemafibrate has been reported to improve NASH development in another diet-induced NASH model, AMLN mice^16^. AMLN mice showed pronounced hepatomegaly and intrahepatic lipid accumulation, but not hypertriglyceridemia possibly due to suppressed hepatic triglyceride secretion by high dietary cholesterol intake^56^. Pemafibrate significantly improves not only liver TG accumulation, but also fasting plasma glucose and insulin levels in AMLN mice. Although precise mechanism of this action is still largely unknown, these observations suggest that pemafibrate potential to improve steatosis in type 2 diabetes patients. Furthermore, these differences on drug responses can be observed in clinically, which reflects on the background differences among patients. Overall, our data combined with previous observations suggest that although PPARα activation reduced macrovesicular steatosis and lipotoxicity via the reduction of excess free fatty acids by enhancing re-esterification and lipid droplet formation, PPARα agonists may not result in a sufficient TG reduction in diabetes-based NAFLD/NASH livers because PPARα is mainly involved in the regulation of nutrient flux to supply glucose and ketone bodies to peripheral tissues, rather than to provide an energy source in the liver. Furthermore, this study suggests that a multidrug intervention against TG accumulation and NAFLD/NASH progression is necessary. In particular, a combination of pemafibrate and drugs that enhance the excretion or inhibit the absorption of carbohydrates and lipids (e.g., an SGLT2 inhibitor, α-glucosidase inhibitor, or pancreatic intestinal lipase inhibitor) has the potential to improve TG accumulation and inflammation in NASH livers.

## Methods

### Chemicals

Pemafibrate was kindly provided from Kowa Co., Ltd. (Nagoya, Japan). Streptozotocin (STZ) was purchased from Sigma Aldrich (MO, USA). Arabic gum was obtained from Wako Pure Chemical Industries (Osaka Japan).

### Animals

Pathogen-free 14-day pregnant C57BL/6J mice were purchased from CLEA Japan (Tokyo, Japan). The STAM mouse NASH model was induced in male mice by a single subcutaneous injection of 200 μg STZ (Sigma, MO, USA) at 2 days after birth and feeding with HFD32 (32%fat, CLEA Japan) ad libitum after 4 weeks of age. Mice at 6 weeks old were randomly divided into two groups: STAM control group fed a HFD32 with vehicle (3% arabic gum in distilled water) treatment and pemafibrate-treated group fed a HFD32 with pemafibrate (0.1 mg/kg in vehicle) treatment for 3 weeks (6–9 weeks). Also, a normal group fed a normal chow (CE-2; 5% fat, CLEA Japan) without STZ injection treated with vehicle for 3 weeks. Pemafibrate or vehicle were given at 5 ml/kg of body weight by oral administration between 0930 and 1000 hours^57^. Mice were sacrificed at 4-hour after the final administration with fasting, and parameters related to fatty liver disease were assessed. All mice were housed in a temperature-controlled (24 °C) facility with a 12-hour light/12-hour dark cycle (0800–2000 hours) and ad libitum access to food and water. The study protocol was approved in accordance with the relevant guidelines and regulations by the Animal Care and Use Committee of the University of Tokyo.

### Blood parameter

Serum levels of total cholesterol, TG, glucose, free fatty acids, AST, ALT, phospholipids, and creatinine were determined using a Labospect 003 autoanalyzer (Hitachi High-Technologies Corporation, Tokyo, Japan).

### Histology

For immunohistochemistry, endogenous peroxidase activity was blocked using 0.03% H_2_O_2_ in Methanol. The sections were incubated with the optimal dilutions of anti-F4/80 (AbD Serotec, Oxford, UK), ER-TR7 (Abcam, Cambridge, MA, USA) antibodies overnight at 4 °C. After incubation with appropriate secondary antibodies, substrate reaction was performed using 3,3′-Diaminobenzidine (Dojindo, Kumamoto, Japan) solution.

NAFLD activity score was calculated according to Kleiner *et al*.^20^. For qualitative assessment of Oil Red O, positive areas were scored for 5 grades by microscopy. For quantitative analysis of F4/80, ER-TR7 and Sirius red positive areas, bright field images of stained sections were captured using a digital camera (DP72, Olympus, Tokyo, Japan) around central veins at 400-fold magnification, and the positive areas in 5 fields were measured using WinROOF image processing software (Mitani, Tokyo, Japan). The results were determined as the means of five different fields of each section.

### RNA-sequencing

The quality of the RNA was assessed by Nanodrop measurement (Thermo Fisher Scientific)^58^. RNA-sequencing (RNA-Seq) libraries were prepared by TruSeq Rapid PE Cluster Kit and TruSeq Rapid SBS kit (Illumina). The libraries were sequenced on Illumina HiSeq. 2500 using a read length of 2 × 150 bp. CASAVA v1.8.2 was conducted and RNA-seq reads were aligned to mouse transcriptome (UCSC gene) and genome (NCBI37/mm9) references respectively using Burrows-Wheeler Aligner. After transcript coordinate was converted to genomic positions, an optimal mapping result was selected either from transcript or genome mapping by comparing the minimal edit distance to the reference. Local realignment was performed within in-house short reads aligner with smaller k-mer size (k = 11). Finally, fragments per kilo base of exon per million fragments mapped (fpkm) values were calculated for each UCSC gene while considering strand-specific information.

### Quantitative real-time PCR (qPCR)

First-strand cDNA was synthesized from total RNA with oligo dT primers using SuperScript III First-Strand Synthesis System (Thermo Fisher Scientific)^59^. The qPCR was performed by SYBR green PCR Master Mix (Thermo Fisher Scientific). The qPCR was carried out in 384-well plates using CFX384 Real-Time system (Bio-Rad). All reactions were performed in triplicate. The relative amount of all mRNAs was calculated using the comparative CT method. Ppib (mouse) and PPIA (human) mRNA were used as the invariant control. Primers used for qPCR are listed in Supplementary Tables S3 and S4.

### Immunoblotting

Harvested liver tissues were homogenated in RIPA buffer (Thermo Fisher Scientific, Tokyo, Japan) supplemented with protease inhibitor cocktail (Sigma-Aldrich, MO, USA). The protein concentration was determined by the Bradford method using protein assay dye reagent concentrate (Bio-Rad, Tokyo, Japan). Whole cell samples were resolved by SDS-polyacrylamide gel electrophoresis, then electro-transferred to nitrocellulose membranes (Bio-Rad, Tokyo, Japan). Membrane were blocked with 5% bovine serum albumin (Wako pure chemical industries, Osaka, Japan) in PBS with 0.1% Tween-20 (Sigma-Aldrich, MO, USA) for 60 min at room temperature. The blot was probed with primary antibody for overnight at 4 °C and then incubated with anti-IgG horse-radish peroxidase-conjugated antibodies for 1 hour at room temperature. Proteins were detected using SuperSignal West Dura Extended Duration Substrate (Thermo Fisher Scientific, Tokyo, Japan) according to the instructions of the manufacturer. Immunoreactive protein bands were documented using a Bio-Rad ChemiDoc XRS + system (Bio-Rad, Tokyo, Japan). Antibodies used for the immunoblot are listed in Supplementary Table S5.

### Lipid droplet analysis

For hepatic lipid droplet analysis, “Image J” imaging software (https://imagej.nih.gov/ij/download.html) was used. H&E staining images were opened in Image J software, and converted into grayscale (8 bit). Then, lipid drop areas were extracted by setting threshold (Min: 220, Max: 255). After excluding blood vessels, lipid droplet areas were analyzed and quantified by “Analyze particles” function. Quantified lipid droplet area data was given by pixel and converted into μm^2^ (1 μm = 3 pixels: determined by scale bar size). Lipid droplet diameter was also calculated. Data were determined as mean values from three different images of each animal. Histogram was created by Microsoft Excel spreadsheet software.

### Statistical analyses

All data are presented as mean ± s.e.m. The homogeneity in variance was evaluated by Bartlett test followed by parametric or non-parametric Dunnett’s multiple comparison test (one-side). The Bonferoni’s multiple comparison test (one-side) was used to compare the data between the STAM control and pemafibrate-treated groups. *P < 0.05, **P < 0.01.

## Supplementary information

Supplementary information.
