# Supplementary material for: Pemafibrate, a selective PPARα modulator, prevents non-alcoholic steatohepatitis development without reducing the hepatic triglyceride content
Source: Sci Rep. 2020 May 8;10:7818. doi: 10.1038/s41598-020-64902-8 (PMC7210999; doi:10.1038/s41598-020-64902-8)
Supplement: Supplementary file 1 — Supplementary information. [file 41598_2020_64902_MOESM1_ESM.docx]

Supplementary information

**Pemafibrate, a selective PPARα modulator (SPPARMα), prevents non-alcoholic steatohepatitis (NASH) development without reducing hepatic triglyceride content**

Yusuke Sasaki, Masato Asahiyama, Toshiya Tanaka, Shogo Yamamoto, Kentaro Murakami, Wakana Kamiya, Yoshihiro Matsumura, Tsuyoshi Osawa, Motonobu Anai, Jean‑Charles Fruchart, Hiroyuki Aburatani, Juro Sakai & Tatsuhiko Kodama


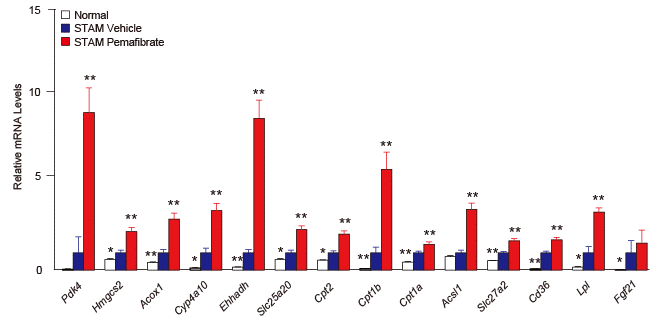


**Supplementary Figure S1.**  **Pemafibrate induce and PPARα target genes in STAM mouse liver.** qPCR validation of PPAR target genes in normal or STAM control or pemafibrate-treated STAM mouse liver (n=6). Primers used for qPCR are listed in Supplementary Table S4. Error bars show s.e.m. **P* < 0.05; ***P* < 0.01.


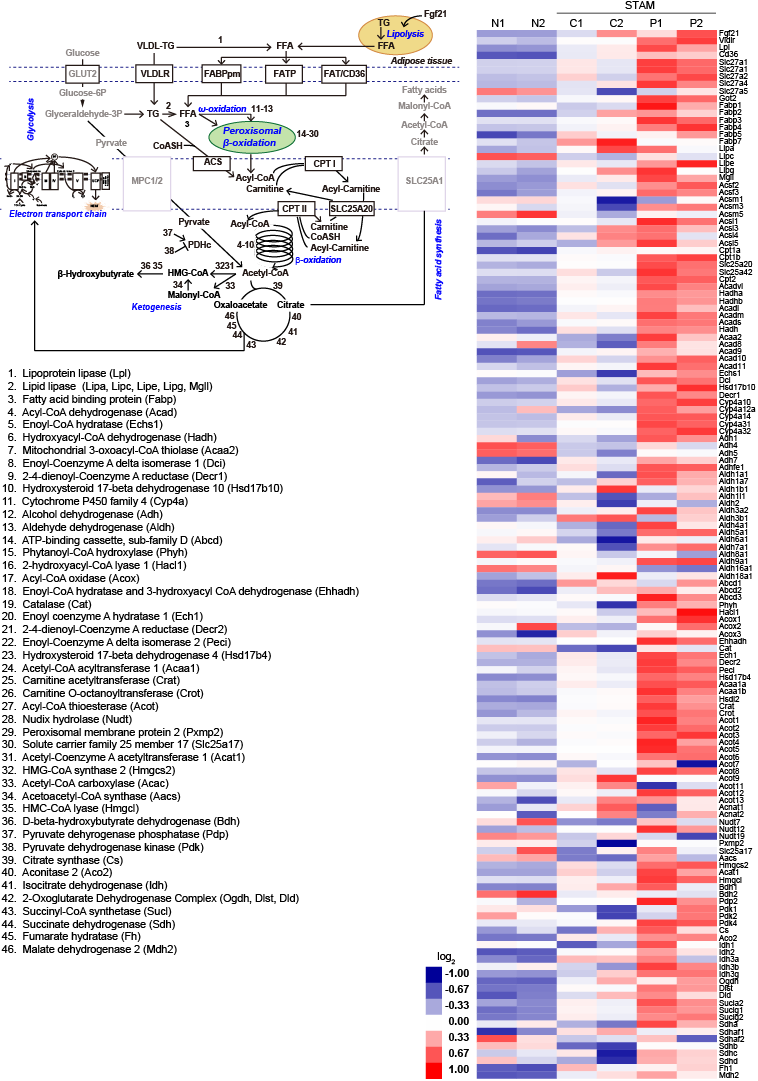


**Supplementary Figure S2. Effect of pemafibrate on fatty acid metabolism-related genes expression in STAM mouse liver.** Schematic representation of the fatty acid metabolism pathways in the liver is shown in left panel. Heat map showing changes in expression of selected glucose metabolism and triglyceride synthesis-related markers in normal (N1 and N2) or STAM control (C1 and C2) or pemafibrate-treated (P1 and P2) STAM mouse liver.


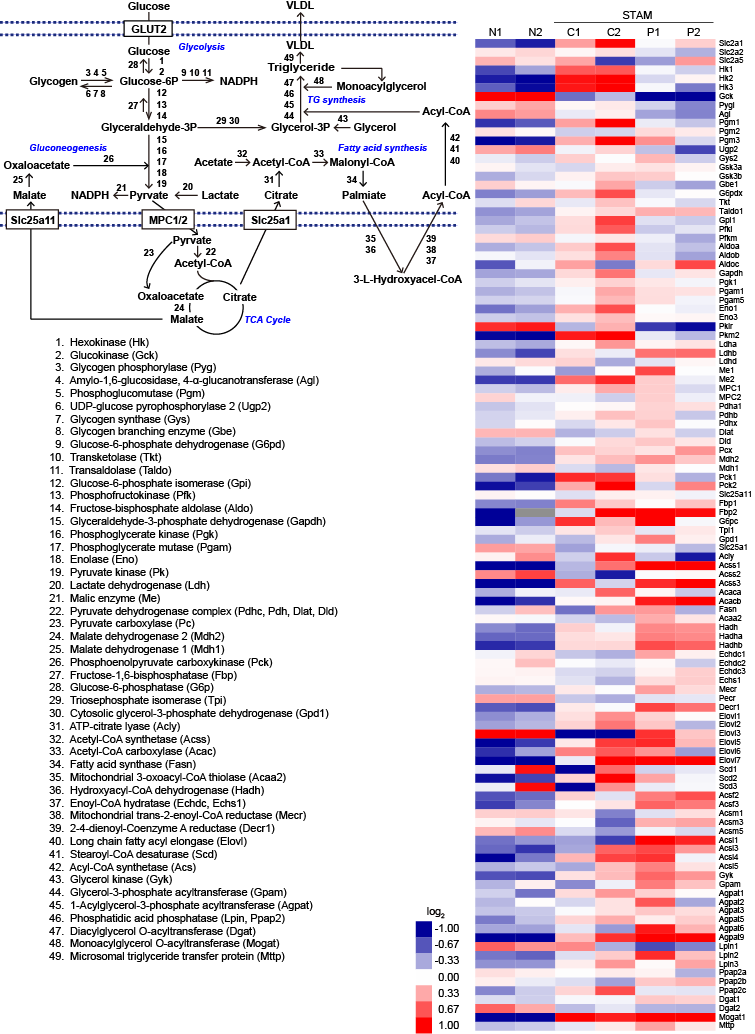


**Supplementary Figure S3. Effect of pemafibrate on glucose metabolism and triglyceride synthesis-related genes expression in STAM mouse liver.** Schematic representation of the glycolytic, gluconeogenic, and triglyceride synthesis pathway in the liver is shown in left panel. Heat map showing changes in expression of selected glucose metabolism and triglyceride synthesis-related markers in normal (N1 and N2) or STAM control (C1 and C2) or pemafibrate-treated (P1 and P2) STAM mouse liver.


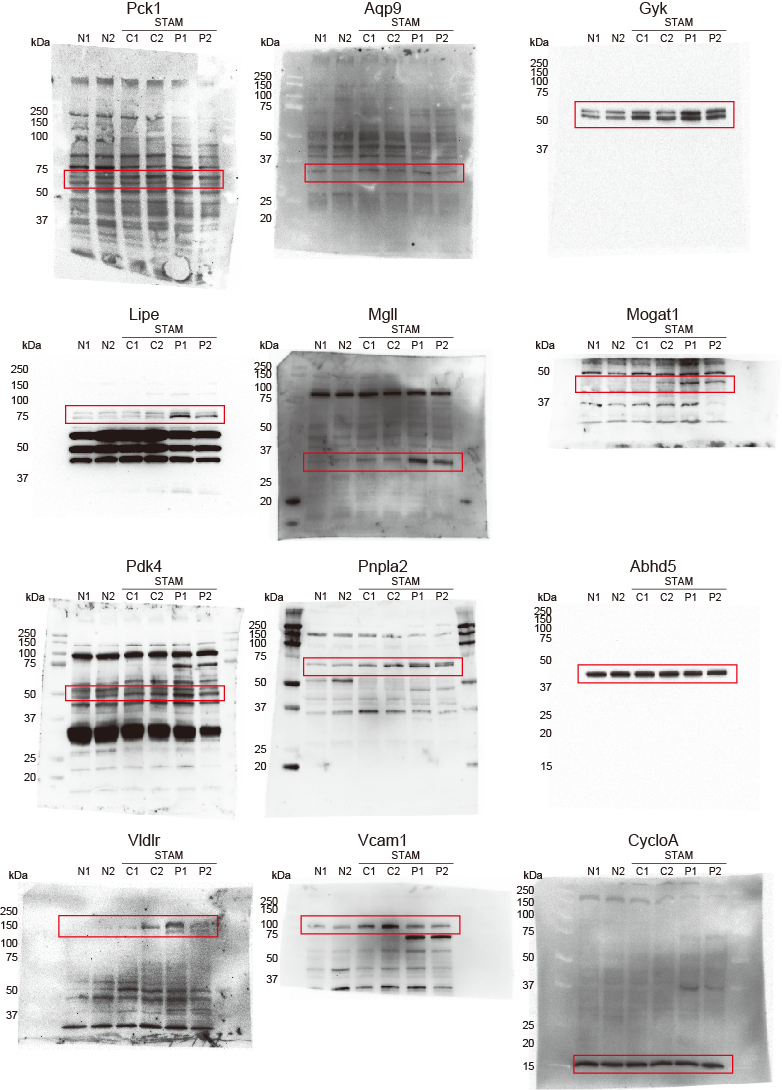


**Supplementary Figure S4. Unprocessed immunoblots for Figures 2 and 3.**

**Supplementary Table S1. RNA-seq analysis of pemafibrate treated NASH mouse Liver**

|  |  | Pemafibrate targets |
| --- | --- | --- |
| Up-regulated genes |  | 187 |
| Down-regulated genes |  | 477 |

Up-regulated genes; FPKM (pemafibrate) ≥ 1, Pemafibrate/Control ratio ≥ 2^0.6^

Down-regulated genes; FPKM (Control) ≥ 1, Pemafibrate/Control ratio ≤ 2^-0.6^

**Supplementary Table S2. Gene Ontology (GO) categories significantly enriched in Pemafibrate regulated genes.**

| GO ID | GO Category name | Total | Changed | LOD | P Value |
| --- | --- | --- | --- | --- | --- |
| Up-regulated genes | |  |  |  |  |
| 0006629 | Lipid metabolic process | 857 | 49 | 0.93 | 9.88E-26 |
| 0044255 | Cellular lipid metabolic process | 632 | 40 | 0.958 | 1.41E-22 |
| 0006631 | Fatty acid metabolic process | 249 | 26 | 1.169 | 3.00E-20 |
| Down-regulated genes | |  |  |  |  |
| 0002376 | Immune system process | 1020 | 123 | 0.817 | 5.25E-50 |
| 0009605 | Response to external stimulus | 1030 | 105 | 0.718 | 9.70E-36 |
| 0002682 | Regulation of immune system process | 840 | 92 | 0.744 | 1.19E-33 |

Gene Ontology (GO) attributes were carried out using web-based tool FuncAssociate 2.1

(Berriz GF et al., Bioinformatics 19, 2502(2003)). LOD: Logarithm (base 10) of the odds ratio.

**Supplementary Table S3. qPCR Primer lists**

| Gene |  | | | Sequence |
| --- | --- | --- | --- | --- |
| *Slc2a2* | Forward | | | 5'- TGAGTTCCTTCCAGTTCGGC -3' |
|  | Reverse | | | 5'- AGCTTTCCGGTCATCCAGTG -3' |
| *Hk1* | Forward | | | 5'- CAGTGTGAAGTCGGCCTGAT -3' |
|  | Reverse | | | 5'- GGCTCCCCATTCCGTGTTAA -3' |
| *Gck* | Forward | | | 5'- CGGATGGTGGATGAGAGCTC -3' |
|  | Reverse | | | 5'- AGCACAAGTCGTACCAGCTC -3' |
| *Gpi1* | Forward | | | 5'- GCTTGTCCCCTGAGACTTCC -3' |
|  | Reverse | | | 5'- CGCTTCGAGAAACCACTCCT -3' |
| *Pfkl* | Forward | | | 5'- GCACTGACATGACCATTGGC -3' |
|  | Reverse | | | 5'- CCGTCCCATCACCTCCAAAA -3' |
| *Aldob* | Forward | | | 5'- CCACGAGACCCTCTACCAGA -3' |
|  | Reverse | | | 5'- TGTTTGTTCCTGCAAGCGGG -3' |
| *Pck1* | Forward | | | 5'- TGCGGATCATGACTCGGATG -3' |
|  | Reverse | | | 5'- AGGCCCAGTTGTTGACCAAA -3' |
| *Tpi* | Forward | | | 5'- CAGCAGGCACAGGAAGTACA -3' |
|  | Reverse | | | 5'- GCTCCAGTCACAGAACCTCC -3' |
| *Gpd1* | Forward | | | 5'- GACCTGATGCAGACACCCAA -3' |
|  | Reverse | | | 5'- AAGCCAAGCCCATCACAGAA -3' |
| *Gpam* | Forward | | | 5'- ACACAAGGCACAGAGGATGG -3' |
|  | Reverse | | | 5'- TTGCCTCTTGGACTCTGCTG -3' |
| *Agpat6* | Forward | | | 5'- CCCTGCCATCTTTGGAGTGT -3' |
|  | Reverse | | | 5'- CCTCTCCTTGGCTCCTCTCT -3' |
| *Agpat9* | Forward | | | 5'- TCCCTATCTGGCACCATCCA -3' |
|  | Reverse | | | 5'- GAGAGGTGTGATTGGCGACA -3' |
| *Lpin2* | Forward | | | 5'- TTTCACGTACGCTTCGGGAA -3' |
|  | Reverse | | | 5'- TCCACAGCACTGCCATTGAT -3' |
| *Ppap2b* | Forward | | | 5'- CAATTGCTCCGAGGGCTACA -3' |
|  | Reverse | | | 5'- AACATGGAGAAGGAGGCGTG -3' |
| *Dgat1* | Forward | | | 5'- CTCAACTTTCCTCGGTCCCC -3' |
|  | Reverse | | | 5'- GATCAGCCCCACTTGAAGCT -3' |
| *Mttp* | Forward | | | 5'- AGAGGACAGCTTTGTCACCG -3' |
|  | Reverse | | | 5'- CTTCCCGGGGATCATCCTTG -3' |
| *Lipe* | | Forward | 5'- TCCAGTTCACACCTGCCATC -3' | |
|  | | Reverse | 5'- GTCACACTGAGGCCTGTCTC -3' | |
| **Supplementary Table S3. qPCR Primer lists (continued)** | | | | |
| *Gene* |  | | | Sequence |
| *Mgll* | Forward | | | 5'- ATCATCCCGGAGTCAGGACA -3' |
|  | Reverse | | | 5'- TTGGTCACTTCCGGAAGCTC -3' |
| *Mogat1* | Forward | | | 5'- ACATCGTATCTCCACGTGGC -3' |
|  | Reverse | | | 5'- CTCCATCCTTGCTCAGCACA -3' |
| *Vldlr* | Forward | | | 5'- TCAACTGCCCTTCTCGAACC -3' |
|  | Reverse | | | 5'- AGCCATCAACACAGTCTCGG -3' |
| *Aqp9* | Forward | | | 5'- CGAGCCAAGAAGAACCTCGT -3' |
|  | Reverse | | | 5'- CCACCAGCCTTTTCTCGACT -3' |
| *Gyk* | Forward | | | 5'- TGGCCTAATGAAAGCTGGGG -3' |
|  | Reverse | | | 5'- AGCACCCTGTTCCATACGTG -3' |
| *Vcam1* | Forward | | | 5'- TCCGTTCTGACCATGGAGCC -3' |
|  | Reverse | | | 5'- TCTGGATCCTTGGGGAAAGA -3' |
| *Marco* | Forward | | | 5'- GAGAGAAGGGCAGCAAAGGT -3' |
|  | Reverse | | | 5'- CTTCATGCCCATGTCCCCTT -3' |
| *Emr1* | Forward | | | 5'- AAACGAGGCTTCCTGTCCAG -3' |
|  | Reverse | | | 5'- CCAGGATATTGGTGCAGACT -3' |
| *Lgals3* | Forward | | | 5'- ACAGTCAGCCTTCCCCTTTG -3' |
|  | Reverse | | | 5'- TCCCCAGTTGGCTGATTTCC -3' |
| *Mmd2* | Forward | | | 5'- CTCTGTGGCCTCTTTGTGGT -3' |
|  | Reverse | | | 5'- CAGCCAAGGAGCATAGGAGG -3' |
| *Cd44* | Forward | | | 5'- ATGAAGTTGGCCCTGAGCAA -3' |
|  | Reverse | | | 5'- GTGTTGGACGTGACGAGGAT -3' |
| *Cyba* | Forward | | | 5'- GGTGTGCTCATCTGTCTGCT -3' |
|  | Reverse | | | 5'- GTAATTCCTGGTGAGGGGCC -3' |
| *Pkm2* | Forward | | | 5'- TCACCCTGGACAACGCTTAC -3' |
|  | Reverse | | | 5'- AGTCAGCGCCTTTCTCCTTC -3' |
| *Cd86* | Forward | | | 5'- GCTTCAGTTACTGTGGCCCT -3' |
|  | Reverse | | | 5'- TTAGAGGCTGTGTTGCTGGG -3' |
| *Mmp12* | Forward | | | 5'- CTTAGAGCAGTGCCCCAGAG -3' |
|  | Reverse | | | 5'- CGCTTCATGTCCGGAGTGTA -3' |
| *Cxcl10* | Forward | | | 5'- CATCCTGCTGGGTCTGAGTG -3' |
|  | Reverse | | | 5'- CAAGCTTCCCTATGGCCCTC -3' |
| *S100a4* | Forward | | | 5'-GCTTCCTGGGGAAAAGGACA -3' |
|  | Reverse | | | 5'- ATGGCAATGCAGGACAGGAA -3' |

**Supplementary Table S3. qPCR Primer lists (continued)**

| Gene | |  | Sequence |
| --- | --- | --- | --- |
| *Ppib* | | Forward | 5'- CAAAGACACCAATGGCTCACAG -3' |
|  | | Reverse | 5'- CCACATCCATGCCCTCTAGAA -3' |
| *PDK4* | | Forward | 5'- GAGGTGGTGTTCCCCTGAGAATT-3' |
|  | | Reverse | 5'- CAAAACCAGCCAAAGGAGCATT -3' |
| *VCAM1* | Forward | 5'- GGGAAGATGGTCGTGATCCTT -3' |  |
|  | Reverse | 5'- TCTGGGGTGGTCTCGATTTTA -3' |  |
| *PPIA* | Forward | 5'- TGGTTCCCAGTTTTTCATCTGC-3' |  |
|  | Reverse | 5'- CCATGGCCTCCACAATATTCA-3' |  |

**Supplementary Table S4 qPCR Primer lists**

| Gene |  | Sequence |
| --- | --- | --- |
| *Pdk4* | Forward | 5'- GAGGTGGTGTTCCCCTGATT -3' |
|  | Reverse | 5'- CAAAACCAGCCAAAGGAGCATT -3' |
| *Hmgcs2* | Forward | 5'- CTAGAGGCCTTCAGGGGTCT -3' |
|  | Reverse | 5'- GGTAAAGGGAGGCCTTGGTC -3' |
| *Acox1* | Forward | 5'- AGAGTCTGTCCAAGGCATGC -3' |
|  | Reverse | 5'- AAAATCTGGGGCTCTGGCTC -3' |
| *Cyp4a10* | Forward | 5'- GTCACCTTCCCTGATGGACG -3' |
|  | Reverse | 5'- CTCTGGATTTGGCCACACCT -3' |
| *Ehhadh* | Forward | 5'- TCCCTCAGGAGCATCTTGGA -3' |
|  | Reverse | 5'- CCCTTGCAAAAGAGATGGCG -3' |
| *Slc25a20* | Forward | 5'- CAATGCAGTCATGATCCGCG -3' |
|  | Reverse | 5'- ACAAGTTGGGGGCAATCCAA -3' |
| *Cpt2* | Forward | 5'- TCAGTGCACAGAAGCCTCTC -3' |
|  | Reverse | 5'- CTCCTTCCCAATGCCGTTCT -3' |
| *Cpt1b* | Forward | 5'- CATGTATCGCCGCAAACTGG -3' |
|  | Reverse | 5'- CCTGGGATGCGTGTAGTGTT -3' |
| *Cpt1a* | Forward | 5'- AGTCCTGCAACTTTGTGCTGGC -3' |
|  | Reverse | 5'- GGTACAGGTGCTGGTGCTTTTCA -3' |
| *Acsl1* | Forward | 5'- GTACGATGGCTTCCAGAGGG -3' |
|  | Reverse | 5'- ACTCATAGGGCTGGTTTGGC -3' |
| *Slc27a2* | Forward | 5'- CATCGTGGTTGGGGCTACTT -3' |
|  | Reverse | 5'- GGTACCGAAGCAGTTCACCA -3' |
| *Cd36* | Forward | 5'- GACGTGGCAAAGAACAGCAG -3' |
|  | Reverse | 5'- ATGGCTCCATTGGGCTGTAC -3' |
| *Lpl* | Forward | 5'- ACTCGCTCTCAGATGCCCTA -3' |
|  | Reverse | 5'- ATCTCGAAGGCCTGGTTGTG -3' |
| *Fgf21* | Forward | 5'- CCTGGAGATCAGGGAGGATGGAA -3' |
|  | Reverse | 5'- TCCATCTGGCTGTTGGCAAAGAA -3' |

**Supplementary Table S5 Antibody lists**

| Antibody | Catalog No. | Source |
| --- | --- | --- |
| Cyclophilin A | #2175 | Cell Signaling Technology |
| Pck1 | #12940 | Cell Signaling Technology |
| Hormone sensitive lipase/HSL | ab45422 | Abcam |
| Monoacylglycerol Lipase/MGL | ab24791 | Abcam |
| VCAM1 | ab134047 | Abcam |
| Glycerol kinase | ab126599 | Abcam |
| Abhd5/CGI-58 | ab183739 | Abcam |
| ATGL | 55190-1-AP | Proteintech |
| PDK4 | 12949-1-AP | Proteintech |
| AQP9 | sc-74409 | Santa Cruz Biotechnology |
| Vldlr | MAB2258 | R&D Systems |
| Mogat1 | AV50240 | Sigma-Aldrich |
| mouse IgG HRP | A4416 | Sigma-Aldrich |
| rabbit IgG HRP | #7074 | Cell Signaling Technology |
| rat IgG HRP | #7077 | Cell Signaling Technology |
